# Management of Small Bowel Neuroendocrine Tumors

**DOI:** 10.3390/cancers11091395

**Published:** 2019-09-18

**Authors:** Vincent Larouche, Amit Akirov, Sameerah Alshehri, Shereen Ezzat

**Affiliations:** 1 Endocrine Oncology Site Group, Princess Margaret Cancer Centre, University of Toronto, Toronto, ON M5G2C1, Canada; amit.akirov@uhn.ca (A.A.); sameerah.alsherhri@uhn.ca (S.A.); shereen.ezzat@uhn.ca (S.E.); 2 Division of Endocrinology and Metabolism, Department of Medicine, Jewish General Hospital, McGill University, Montreal, QC H3T1E2, Canada; 3 Department of Medicine, Institute of Endocrinology, Beilinson Hospital, Petach Tikva 4941492, Israel; 4 Department of Medicine, Sackler School of Medicine, Tel Aviv University, Tel Aviv 6997801, Israel

**Keywords:** small bowel, neuroendocrine tumor, carcinoid, carcinoid syndrome, neuroendocrine carcinoma, somatostatin analogue, everolimus, PRRT

## Abstract

Several important landmark trials have reshaped the landscape of non-surgical management of small bowel neuroendocrine tumors over the last few years, with the confirmation of the antitumor effect of somatostatin analogue therapy in PROMID and CLARINET trials as well as the advent of therapies with significant potential such as mammalian target of rapamycin inhibitor (mTor) everolimus (RADIANT trials) and peptide receptor radionuclide therapy (PRRT) with 177-Lutetium (NETTER-1 trial). This narrative summarizes the recommended management strategies of small bowel neuroendocrine tumors. We review the main evidence behind each recommendation as well as compare and contrast four major guidelines, namely the 2016 Canadian Consensus guidelines, the 2017 North American Neuroendocrine Tumor Society guidelines, the 2018 National Comprehensive Cancer Network guidelines, and the 2016 European Neuroendocrine Tumor Society guidelines. Different clinical situations will be addressed, from loco-regional therapy to metastatic unresectable disease. Carcinoid syndrome, which is mostly managed by somatostatin analogue therapy and the serotonin antagonist telotristat etiprate for refractory diarrhea, as well as neuroendocrine carcinoma will be reviewed. However, several questions remain unanswered, such as the optimal management of neuroendocrine carcinomas or the effect of combining and sequencing of the aforementioned modalities where more randomized controlled trials are needed.

## 1. Introduction

Neuroendocrine tumors (NETs) are a heterogeneous group of neoplasms arising from various anatomic sites, the majority originating from the small bowel or pancreas. A recent SEER (Surveillance, Epidemiology and End Results) database study showed that the age-adjusted incidence of NETs has risen to 6.98 per 100,000 per year in the US in 2012 [1]. This rise in incidence is thought to be due, at least partly, to increased incidental findings on imaging and screening endoscopy for colorectal polyps or cancer. NETs usually have an indolent clinical course, but tumor behavior varies widely depending on 2017 WHO (World Health Organization) grading [2], based on Ki-67 proliferation index and mitotic count. Extent of disease, defined by AJCC (American Joint Committee on Cancer) staging, including tumor size and invasion, presence or absence of locoregional lymph nodes or metastases is also a paramount prognostic indicator [3]. Five-year survival is estimated to be around 85% for small bowel NETs with an overall median survival of 9.3 years [4].

Most small bowel neuroendocrine tumors are enterochromaffin cell tumors that make serotonin; however, because of the vascular drainage, the serotonin is inactivated in the liver, therefore these tumors tend to be hormonally inactive until they develop extensive liver metastases, when the excess serotonin gives rise to carcinoid syndrome, a constellation of symptoms including flushing, diarrhea and right-sided heart failure. This differs from the clinical presentation of their pancreatic counterpart (pancreatic neuroendocrine tumors, pNET), where symptoms related to hormone over-secretion by the tumor can occur with even small localized tumors. Examples of typical functional pNET clinical syndromes include excess insulin (insulinoma) leading to recurrent hypoglycemic episodes, gastrin excess (gastrinoma) giving rise to Zollinger-Ellison syndrome, glucagon excess (glucagonoma) leading to diabetes, diarrhea, necrolytic erythema migrans and venous thrombosis, vasoactive intestinal peptide (VIPoma) leading to diarrhea, achlorhydria and hypokalemia (Verner–Morrison syndrome) somatostatin (somatostatinoma) leading to diabetes, diarrhea and gallstones, or pancreatic polypeptide (PPoma) leading to diarrhea. Although so-called “non-functional pNETs” can secrete several peptides, such as chromogranin, the symptoms in these cases are nonspecific such as abdominal pain or weight loss and they tend to be diagnosed at a later stage with a larger primary tumor and more often metastatic [5,6].

As outlined in recent review articles [5,6,7], over the last decade, several important landmark trials have reshaped the management of small bowel neuroendocrine tumors [8,9,10,11]. NETs are rare neoplasms and their management requires investigation, treatment and follow-up by multidisciplinary teams in specialized centres. This narrative summarizes the recommended management strategies of small bowel neuroendocrine tumors in different clinical situations. We review the main evidence behind each recommendation as well as compare and contrast four major guidelines, namely the 2016 Canadian Consensus guidelines [12], the 2017 North American Neuroendocrine Tumor Society guidelines (NANETS) [13,14], the 2018 National Comprehensive Cancer Network guidelines (NCCN) [15] and the 2016 European Neuroendocrine Tumor Society guidelines (ENETS) [16,17].

## 2. Locoregional Disease

**Figure 1:**
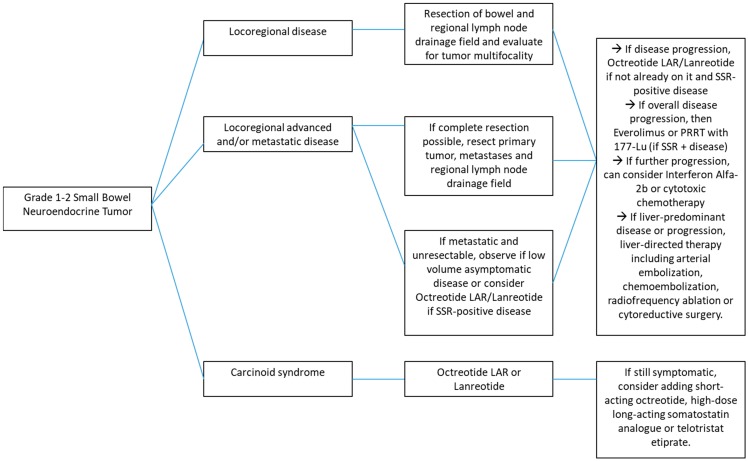
Management of Grade 1–2 Small Bowel Neuroendocrine Tumors—Flow diagram.

All major guidelines [12,13,14,15,16,17] concur that any locoregional small bowel NET should be resected en bloc with its lymphatic drainage field, including the mesentery. Table 1 and Figure 1 summarize all guidelines recommendations. This is due to the fact that small bowel neuroendocrine tumors have a significant metastatic potential, even at a size less than 2 cm. Rorstad et al [18] compiled data in a systematic review showing that small bowel neuroendocrine tumors (NETs) had nodal and distant metastases at diagnosis, respectively, in 12% and 5% for tumors less than 1 cm, 70% and 19% in tumors ranging from 1.0 to 1.9 cm, 85% and 47% in tumors greater than 2.0 cm.

As up to 40% of small bowel NETs may have more than one site of primary gastrointestinal (GI) tract malignancy, the entire small and large bowels should be evaluated both preoperatively and intraoperatively. Both the ENETs [16,17] and NANETs [13,14] guidelines favor open resection as the preferred surgical approach, although both mention that laparoscopic resection is an option if the bowel can be “run through”, that is, examined thoroughly by palpation, via a small incision. As for the NCCN [12] and Canadian consensus guidelines [12], emphasis is put on the need to evaluate the possibility of tumor multifocality intraoperatively, but the authors did not favor any surgical approach over the other. Canadian consensus guidelines [12] mention that positive margins or residual disease after initial resection of primary tumor warrants a definitive resection, however other guidelines did not specifically address this situation. A retrospective study by Cives et al. of 129 midgut NET patients (Stage I–III) who underwent surgical resection showed recurrence rates of 0%, 35.7% and 31.7%, respectively, for stage I, II and III tumors with a median follow-up time of 81 months [19].

Overall, this recommendation for en bloc resection of any primary small bowel NET with its lymphatic drainage field is consistent across all major guidelines [12,13,14,15,16,17] and with current evidence. In terms of surveillance after a surgical resection for curative intent, NCCN guidelines [15] recommend follow-up biochemical markers and imaging (abdomen-pelvis +/− chest cross-sectional imaging) at 3–12 months post resection and then every 12–24 months for up to 10 years, NANETS guidelines [13,14] advocate for yearly imaging, ENETS guidelines [16,17] recommend imaging every 6–12 months except for Grade 3 tumors (every 3 months) and Canadian guidelines [12] only recommend regular imaging without detailing frequency.

## 3. Synchronous Primary and Metastatic Disease

In small bowel NETs, the liver is the most common site of metastasis. When metastatic disease is present, it is necessary to establish if the patient has carcinoid syndrome, usually by measuring 5-HIAA (5-hydroxyindolacetic acid) a metabolite of serotonin, in a 24 h urine collection. As carcinoid syndrome is usually associated with extensive unresectable metastatic disease, surgery has a limited role in this situation.

As opposed to most malignancies where there are no or few advantages to resecting the primary tumor in the setting of metastatic disease, small bowel neuroendocrine tumors are often associated with desmoplastic reaction and fibrosis and thus intermittent small bowel obstruction or even ischemia. Guidelines [12,13,14,15,16,17] thus unanimously recommend that in the setting of resectable synchronous primary tumor and hepatic metastases, resection of the primary tumor(s), lymph node drainage field in combination with liver metastases is warranted. The practice of resecting an asymptomatic small bowel NET primary in the setting of unresectable metastatic disease remains controversial and both ENETS [16,17] and NANETS [13,14] guidelines acknowledge that the lack of prospective evidence does not permit definite conclusions on any potential survival benefit and that in this case, risks and benefits of the surgical intervention need to be considered. Current evidence on the benefits of resecting a small bowel NET primary in the presence of unresectable hepatic metastases is conflicting. Capurso et al. [20] conducted a systematic review and found that patients who underwent surgical resection of a small bowel NET primary with unresectable liver metastases had an overall survival ranging from 75 to 139 months compared to 50 to 88 months in those who were not operated on. However, they could not perform a meta-analysis and the difference was statistically significant in only 3 out of 6 studies prone to selection bias of retrospective reporting. More recently, Almond et al. [21] conducted a meta-analysis looking at the impact on overall survival following palliative surgery to remove the primary lesion in unresectable metastatic small intestinal and pancreatic NETs. Their data demonstrated, via an analysis of pooled multivariate hazard ratios, a significantly longer overall survival in patients undergoing resection of small bowel NETs (HR 0.47, 0.35–0.55, *p* = 0.007). In fact, increase in median survival in patients treated surgically versus non-surgically ranged from 22 to 112 months in small bowel neuroendocrine tumors and the number needed to treat (NNT) to have one more patient alive at five years ranged from 1.7 to 7.7. Conversely, Daskalakis et al. [22] conducted a cohort study in Sweden comparing patients with metastatic small bowel neuroendocrine tumors; 161 patients underwent up-front locoregional surgery and 202 underwent delayed surgery (meaning more than 6 months after diagnosis), both received systematic oncologic therapy for their neuroendocrine tumors. Overall, there was no survival advantage to up-front locoregional surgery as compared to delayed surgery and patients in the delayed group required fewer reoperations.

Although evidence and guidelines [12,13,14,15,16,17] are clear as to recommend resection of a primary small bowel NET and liver metastatic foci when feasible, as mentioned above, the data are rather conflicting when it comes to resecting a primary NET in the setting of unresectable metastatic disease, where it becomes a case by case discussion. Further research, including prospective trials comparing surgical resection vs. conservative management of primary small bowel NET with unresectable metastatic disease, is needed.

## 4. Hepatic Metastases

Most current NET guidelines [12,13,14,15,16,17] recommend surgical resection of hepatic metastases associated with small bowel neuroendocrine tumor. This is particularly relevant in cases where there is absence of diffuse bilobar liver involvement, compromised liver function, or Grade 3 tumor (neuroendocrine carcinoma). Canadian consensus guidelines [12], ENETS [16,17] and NCCN guidelines [15] indeed recommend resection of liver metastases with the goal of preserving liver parenchyma and both left and right inflow and outflow vascular patency when possible. For limited disease, particularly hepatic metastases measuring less than 3 cm, they recommend image-guided ablation alone or in combination with surgery. Similarly, the NANETS guidelines [13,14] advocate that hepatic metastases should be resected when anatomically feasible and with low mortality and morbidity. They recommend parenchymal-sparing procedures and specify that patients with any number or size of metastases, intermediate grade or extrahepatic disease should be considered candidates for liver debulking operations if a 70% debulking threshold can be achieved. If cytoreductive/ablative procedures are contra-indicated, most guidelines recommend bland embolization, chemoembolization or radioembolization with no clear advantage of one form over the others. ENETS guidelines [16,17] also mention that selective internal radiation therapy is another option for management of inoperable hepatic metastases in a clinical trial setting.

The basis for these recommendations are retrospective cohort studies [23,24,25,26,27,28,29,30,31,32] where the 10-year survival rate was around 50–60% in patients with small bowel neuroendocrine tumors who underwent either surgical resection or ablation of liver metastases. Symptomatic improvement was also noted. That being said, these were retrospective studies prone to selection bias.

Mayo et al. [33] compared outcomes in 339 patients who underwent surgical resection of hepatic metastasis of a neuroendocrine tumor (small bowel or pancreatic primary) to 414 patients who were managed with intra-arterial therapy. These authors used propensity index modeling to address the aforementioned biases. In their analysis, surgery was associated with better outcomes in patients with a low (< 25%) liver disease burden and in those who were symptomatic with > 25% liver involvement. However, there was no difference in long-term outcome in asymptomatic patients with high liver disease burden (> 25%) and, therefore, a non-surgical and less-invasive approach was deemed a more appropriate management strategy for that subgroup of patients. In the same stream of thought, Norlén et al. [34] compared 103 patients who underwent surgical resection or ablation therapy for liver metastases of small bowel NETs to 273 controls whose liver metastases were not treated surgically. They also used propensity score matching, which resulted in two groups of 73 patients each with similar baseline characteristics. The 5-year overall survival was similar at 74% in both groups. The difference in results likely stems from the fact the study by Mayo et al. [33] compared two different treatment modalities (surgical resection vs. intra-arterial therapy) for neuroendocrine tumor liver metastases whereas Norlén et al. [34] compared active therapy by surgery and/or radio-frequency ablation to observation.

In terms of radio-frequency ablation specifically, Akyldiz et al. [35] published the largest prospective cohort study to date. Eighty-nine patients with a NET (55 small bowel primary, 23 pancreatic, 11 medullary thyroid cancer) were followed for a median follow-up time of 30 months. The majority (97%) experienced symptomatic relief after RFA, 22% developed local recurrence, 63% developed new liver lesions, and 59% developed extrahepatic disease during follow-up. Median disease-free survival was 1.3 years and the overall survival was 6 years after RFA. As most patients present with multifocal and bilateral disease, a primary role for ablation may be as an adjunct to surgical resection to allow local treatment of all disease when hepatectomy alone might compromise residual liver function. Ablation may also be particularly useful for patients with hepatic disease recurrence in whom surgical options are limited due to prior hepatectomy.

Hepatic artery embolization is based on the principle that liver tumors derive most of their blood supply from the hepatic artery as opposed to healthy hepatocytes, which are mostly supplied by the portal vein. Given that there are no randomized controlled trials, any technique between bland embolization, chemoembolization, or radio-embolization is a reasonable approach for patients with small bowel NETs and hepatic-predominant disease who are not surgical candidates. Overall, response rates associated with all of these techniques, either decrease in hormone secretion, symptomatic benefit, or radiographic regression, are generally over 50%, even in those with significant hepatic tumor burden [36,37,38].

Kennedy et al. [39] conducted a systematic review of 18 studies comparing trans-arterial embolization, chemoembolization, and selective internal radiation therapy for patients with liver metastases of neuroendocrine origin. In bland or chemoembolization studies, objective radiological response rates varied from 11% to 100% with a median survival ranging between 18 to 80 months. Conversely, in radio-embolization studies, objective radiological response rates varied from 22–71% and median survival durations varied from 22 to 70 months.

Complications of embolization procedures include pain, nausea, fatigue, biochemical abnormalities such as liver enzymes elevation or post-embolization syndrome, defined as fever without sepsis, right upper quadrant pain and possibly nausea and/or vomiting following embolization. Pre-medication including oral and intravenous analgesics, steroids, antibiotics and serotonin receptor antagonists can reduce the severity of such symptoms.

Generally, there is a consensus in current guidelines [12,13,14,15,16,17] that liver metastases of small bowel neuroendocrine tumors should be surgically resected in absence of diffuse bilobar liver involvement, compromised liver function, or Grade 3 tumor (neuroendocrine carcinoma). However, when surgery is not feasible, there is little evidence to guide clinicians on management of such metastases. There is a need for larger prospective studies comparing survival outcomes and symptomatic relief after different treatment modalities including bland embolization vs. chemoembolization vs. radio-frequency ablation of inoperable metastatic liver disease.

## 5. Other Surgical Considerations

With regards to peritoneal metastases, both Canadian consensus guidelines [12] and NANETS guidelines [13,14] recommend surgical resection, where feasible and possibly synchronous resection of peritoneal and hepatic metastases. NANETS guidelines [13,14] also advised against use of HIPEC (hyperthermic intraperitoneal chemotherapy) due to the lack of evidence supporting its use in this disease. Canadian consensus guidelines [12] recommend considering cytoreductive surgery of abdominal disease in select patients with extrahepatic metastases for symptom control. Other guidelines did not specifically address these issues.

Gallstones are a common side effect of chronic somatostatin analogue therapy, occurring in 18–27% patients as per product monographs and 36.6–63% in retrospective surgical series [40,41,42]. NANETS [13,14], ENETS [16,17] and NCCN guidelines [15] recommend prophylactic cholecystectomy if long-term treatment with a somatostatin analogue is anticipated, therefore not in locoregional disease cases. Canadian consensus guidelines [12] go further and recommend considering a prophylactic cholecystectomy at the time of any abdominal surgery performed on a patient with a small bowel NET, including locoregional disease resection. It is important to acknowledge that these are expert recommendations with limited low-quality evidence. More prospective studies are needed to compare outcomes after gallbladder resection versus conservative management.

Orthotopic liver transplantation (OLT) remains an investigational treatment option for unresectable neuroendocrine liver metastases. Canadian consensus guidelines [12] suggest that in cases of a resected Grade 1 primary small bowel neuroendocrine tumor with hepatic only metastases and no disease progression over a minimum 12-month period, liver transplantation may be an option. Similarly, NANETS guidelines [13,14] mention that, although controversial, OLT may be an option if Milan and ENETS criteria are met. Taking into account that such patients usually have a very good prognosis and natural history without OLT, we emphasize that transplantation remains a controversial treatment option, mostly in the setting of a clinical trial. Le Treut et al. [43] in a retrospective multicenter European study described a series of 213 patients from 35 centres who underwent OLT for unresectable liver metastases of a NET primary. At 5 years post-OLT, overall survival was 52% and disease-free survival was 30%. Similarly, Gelady et al. [44] reported a series of 150 patients who underwent OLT for hepatic metastases of NET from the UNOS (United Network for Organ Sharing) database. Their overall survival rates were 81%, 65% and 49% at 1-, 3- and 5- years respectively. Outcomes did not differ with the primary location of the NET and were overall similar to outcomes in OLT for hepatocellular carcinoma. Prospective studies looking at survival outcomes after OLT for liver metastases of small bowel neuroendocrine tumors vs. usual liver-directed therapies are needed.

## 6. Systemic Therapy for Metastatic or Unresectable Disease

Somatostatin analogue therapy is the first line systemic therapy unanimously recommended by all four guidelines in the setting of unresectable, asymptomatic, somatostatin-receptor positive, well-differentiated gastroenteropancreatic NET and a high tumor burden. In the case of small volume disease, observation alone is appropriate and somatostatin analogue therapy can be initiated if there is evidence of clinically meaningful tumor progression.

The mechanisms by which SSA (somatostatin analogues) control tumor growth may result from direct antiproliferative effects, including cycle inhibition and pro-apoptotic effects as well as indirect antiproliferative effects, namely inhibition of tumor angiogenesis and release of trophic hormones. These analogues exert their effect by binding to somatostatin receptors (mostly SSTR-2 and 5) expressed by most NETs.

Two landmark trials confirmed the antiproliferative effect of SSA therapy in gastroentero-pancreatic NET patients, as outlined in Table 2. In 2009, Rinke et al. [8] published the results of the German PROMID trial, a phase IIIB randomized controlled trial comparing outcomes in 85 patients with well-differentiated metastatic midgut NETs, of which 43 received placebo and 42 received octreotide LAR (Long Acting Release formulation) injections. The time to tumor progression was significantly longer in the octreotide-treated patients (median 14.3 months vs. 6 months in the placebo arm) and stable disease was more common in the treated arm (66.7% vs. 33.2% in the placebo arm). Survival analysis was inconclusive given a low number of deaths that occurred during the study period.

In 2014, Caplin et al. [9] presented the results of the European CLARINET trial, a randomized controlled trial comparing outcomes in 204 patients with advanced NETs (Ki-67 < 10%), of which 101 received Lanreotide and 103 received placebo. Median study-drug exposure was 24 months in the Lanreotide arm and 15 months in the placebo arm. During the study period, progression-free survival was not reached in the Lanreotide arm and was 18 months in the placebo arm. Progression-free survival rates at 24 months were higher in the Lanreotide arm (65.1% vs. 33% in the placebo arm). HR for death or progression was 0.47. Again, no difference was noted between groups for overall survival.

Of note, the study population in CLARINET differed from the one in PROMID in that they included tumors from the pancreas, midgut, hindgut or unknown primary and the Ki-67 had to be less than 10%. In PROMID, all patients had midgut tumors only and most patients (97.6% in the treatment arm and 93% in the placebo arm) had KI-67 index < 2%. As the inclusion criteria for the study populations for these landmark trials were quite narrow, larger studies including patients with lung, pancreatic and GI tract NETs with a wider range of Ki-67 indices would shed more light on the antiproliferative effect of somatostatin analogues. Moreover, head-to-head trial comparisons between the different somatostatin analogs would help direct drug selection.

Common side effects of somatostatin analogue therapy include mild nausea, abdominal discomfort, bloating, loose stools and fat malabsorption in one third of patients, which tend to subside over time. Mild glucose intolerance may occur, usually transiently. Up to 63% of patients develop gallstones or biliary sludge with chronic therapy [40,41,42].

For second line therapy, while Canadian consensus guidelines [12] suggest everolimus either as a single agent or combined with somatostatin analogues or peptide receptor radionuclide therapy (PRRT), NANETS guidelines [13,14] preferred PRRT over everolimus and also listed Interferon Alpha 2b or locoregional therapy as less favored options. NCCN [15] and ENETS guidelines [16,17] recommend somatostatin analogue therapy if patients were managed with active surveillance prior or everolimus, PRRT, locoregional therapy, Interferon alpha 2b as second-line therapy options and NCCN guidelines mention cytotoxic chemotherapy as a possibility if no other options are feasible.

Everolimus is an inhibitor of the mammalian target of rapamycin (mTOR) used as a systemic therapy in lung and gastroenteropancreatic neuroendocrine tumors [45]. In 2016, Yao et al. [10] examined the effect of everolimus on 302 patients with advanced well-differentiated lung or gastroenteropancreatic NET in the randomized controlled RADIANT-4 trial. Overall, 205 patients received everolimus 10 mg die and 97 patients received placebo. Progression-free survival was significantly longer in the treatment arm (11 months vs. 3.9 months in the placebo arm). HR for death or progression was 0.48. A noteworthy randomized clinical trial that preceded and led the way was the RADIANT-2 trial, where 211 patients with a Grade 1 or 2 NET received everolimus 10 mg die and 204 received placebo, all of them also received a backbone therapy of octreotide LAR 30 mg intra-muscularly every 4 weeks. Although the progression-free survival was significantly longer in the everolimus + octreotide group (16.4 months vs. 11.3 months in the placebo + octreotide group), the *p* value was higher than the pre-determined boundary at final analysis, therefore missing statistical significance (*p* 0.026 for a pre-determined significance threshold of 0.0246). Nonetheless, the results of RADIANT-2 still serve as the basis for the use of combined everolimus + somatostatin analogue therapy in patients with NET and carcinoid syndrome [46]. An interesting avenue for research would be a prospective trial with three arms comparing progression-free survival in NET patients treated with somatostatin analogues alone vs. everolimus alone vs. combination everolimus + somatostatin analogues. This may help clinicians better guide their patients on the best sequence and combination of systemic therapies in metastatic small bowel NET cases.

A common side effect of everolimus is hyperglycemia and the rate is higher in patients with underlying diabetes mellitus or pre-diabetes. The extent to which this complication is further influenced by somatostatin analogues remains unclear. Although rare, another serious adverse effect of everolimus is pneumonitis; therefore, cough and dyspnea need to be closely monitored during administration of this agent.

Another significant breakthrough on the treatment of well-differentiated metastatic NET occurred with presentation of the NETTER-1 trial by Strosberg et al. in 2017 [11]. Peptide receptor radionuclide therapy (PRRT) uses delivery of targeted radiotherapy to malignant neuroendocrine tumor cells that express somatostatin receptors to cause tumor shrinkage [45]. In NETTER-1, 116 metastatic midgut NET patients received PRRT with ^177^Lu-Dotatate and all 229 patients received a backbone of octreotide LAR (30 mg every 4 weeks in the treatment group as opposed to a higher dose of 60 mg in the control group, which is not a standard regimen). To be included, patients had to have a metastatic or unresectable locally advanced midgut NET that had progressed after at least 12 weeks of treatment with octreotide LAR. Progression-free survival after 20 months was significantly longer in the PRRT + octreotide arm than the octreotide alone arm (65.2% vs. 10.8%) Response rate was 18% in the PRRT arm (vs. 3% in the control arm) and in the interim analysis, fewer deaths had occurred in the PRRT arm (14 vs. 26 in the control group). This is the first trial where a treatment modality was associated with preliminary evidence of an overall survival benefit as seen in an interim analysis. In an interim update at the 2018 ASCO meeting, Strosberg et al. reported that the median overall survival was 27.4 months in the octreotide arm and not reached in the PRRT + octreotide arm [47]. Similarly, the progression-free survival showed 30 events in the treatment arm and 78 in the control arm, with a significant hazard ratio of 0.21. Potential rare toxicities from administration of ^177^Lu-Dotatate include myelosuppression and nephrotoxicity.

For the PROMID, RADIANT-4 and NETTER-1 trials, specific follow-up studies of the cohorts examined health-related quality of life. A recent follow-up study by Rinke et al. [48] showed that for the PROMID trial, patients in the octreotide long-acting arm had fewer and later deteriorations compared to those in the placebo arm and that health-related quality of life was maintained or improved for symptoms such as fatigue, insomnia, diarrhea and pain. For participants in the RADIANT-4 trial, a follow-up study by Pavel et al. [49] showed that there was no significant difference in time to definitive deterioration in those treated with everolimus vs. placebo. The NETTER-1 study group [50] recently published their health-related quality of life data and they showed that those who received 177-Lu had a significant quality of life benefit compared to those treated with octreotide alone, including global health status, physical functioning, role functioning, fatigue, pain, diarrhea, disease-related worries and body image. Unfortunately, similar health-related quality of life measures have not been reported so far for CLARINET trial participants.

Neuroendocrine neoplasms express interferon receptors and binding of this molecule to the receptor stimulates T cells, induces cell cycle arrest and inhibits angiogenesis, resulting in antitumor effect. Multiple retrospective studies [51,52,53,54,55,56,57,58,59,60,61,62,63] have examined the outcomes of administering low-dose interferon alpha to patients with metastatic neuroendocrine tumors. Overall, symptoms of hormone hypersecretion decreased in 40–70% patients and there was radiological tumor stabilization in 40–70% of patients and tumor shrinkage in 20–40% of patients, respectively. When combining somatostatin analogs with interferon injections, different studies have conflicting results. Kolby et al. [64,65,66] conducted a prospective trial with 68 patients with metastatic midgut NET who had surgery and liver-directed therapy prior to enrollment; 35 patients were on octreotide alone as opposed to 33 in the octreotide + interferon group. Although there was no significant difference between groups with regards to survival outcomes, patients in the combination group were noted to have a significantly reduced risk of tumor progression. Contrary to these findings, Arnold et al. [65] conducted a randomized clinical trial on 105 patients with metastatic foregut and midgut NETs in which 51 received octreotide monotherapy and 54 received octreotide + interferon. In that study, combination treatment was not superior to monotherapy concerning progression-free and long-term survival. Interferon is administered subcutaneously 3 to 7 times a week and common side effects include fatigue, depression, myelosuppression, flu-like symptoms, weight loss and alteration of thyroid function. Given this unfavorable side effect profile, Interferon remains a potential therapeutic modality for patients with metastatic small bowel neuroendocrine tumors that have failed somatostatin analogue therapy, but is a generally less favored option across all the major guidelines (NANETS [13,14], ENETS [16,17], NCCN [15]).

Other systemic therapies that have been studied in metastatic small bowel neuroendocrine tumors include the tyrosine kinase inhibitors sunitinib [66], sorafenib [67] and pazopanib [68,69]. In phase 2 trials, these molecules produced low response rates but promising rates of disease stabilization and progression-free survival. Yao et al. [70] conducted a large randomized trial of 427 patients comparing adding VEGF inhibitor bevacizumab vs. Interferon to a backbone therapy of octreotide. Although there was a higher radiologic response rate in the bevacizumab group (12% vs. 4% in the interferon group), no significant differences in progression-free survival were observed.

Cytotoxic chemotherapy is a last resort option for metastatic small bowel NET patients that have failed somatostatin analogue therapy and other systemic therapies such as Everolimus or PRRT. NCCN guidelines [15] suggest capecitabine, dacarbazine, fluorouracil (FU) and temozolomide as potential therapies for those patients in whom no other treatment options are available. Dacarbazine, Etoposide, Paclitaxel, Docetaxel, Gemcitabine, Capecitabine, Streptozocin + FU + doxorubicin + cyclophosphamide, Streptozocin + FU + cyclophosphamide, Streptozocin + FU + cisplatin, temozolomide + thalidomide, gemcitabine + oxaliplatin have all been studied in phase II trials with low objective radiologic tumor response rates and median overall survival varying between 10.5 to 31.5 months [71,72,73,74,75,76,77].

Older randomized trials that generally involved mixed groups of NETs have studied respectively Streptozocin + cyclophosphamide vs. streptozocin + FU, Doxorubicin vs. Streptozocin + FU, Doxorubicin + FU vs. Streptozocin + FU and in all treatment groups, objective radiologic tumor response rate varied between 16–33% and median overall survival varied from 11.1 to 24.3 months [72,76,78].

The combination of capecitabine and temozolomide (CAP-TEM) in patients with metastatic well-differentiated NET has been investigated in two small studies. Fine et al. [79] completed a retrospective study with 18 patients with liver metastases of neuroendocrine tumors who had failed somatostatin analogue therapy, previous chemotherapy and liver-directed therapies. In their study, one patient achieved a complete response (CR), 10 patients achieved a partial response, and 4 patients had stable disease (SD), with an overall total response rate of 61%, a median progression-free survival of 14.0 months and a median overall survival from diagnosis of liver metastases of 83 months. The same group carried out a phase II trial and preliminary results [80] outlined that among 28 patients with metastatic NET who failed somatostatin analog therapy, overall response rate was 43% with stable disease rate of 54%. Specifically, overall response rate was 41% in small bowel NETs. Ongoing median progression-free survival is > 20 months and ongoing median overall survival was > 25.3 months. Although these results appear promising, larger prospective randomized controlled trials are needed to better assess the efficacy of CAP-TEM in small bowel NETs.

With regards to surveillance in cases of advanced or metastatic small bowel NET treated with noncurative modalities, NCCN guidelines [15] recommend biochemical markers and abdominal-pelvic +/− chest cross-sectional imaging every 3–12 months, NANETS guidelines [13,14] recommend imaging every 6–12 months if stable disease, ENETS guidelines [16,17] recommend imaging every 3–6 months (except every 3 months for Grade 3 disease) and Canadian guidelines [12] only recommend regular imaging without precising frequency.

Future treatment avenues include combining the different treatment modalities discussed above for refractory disease. For instance, a recent German cohort study by Yordanova et al. in 15 patients with Grade 2/3 NET who failed either chemotherapy or PRRT alone found that the combination of PRRT with 177-lutetium and capecitabine-temozolomide led to disease control in 38–55% of patients, depending on the imaging modality used. The mean PFS was 7.1 months and the overall survival was 25.3 months in the cohort [81].

## 7. Carcinoid Syndrome and Carcinoid Heart Disease

Carcinoid syndrome refers to a plethora of symptoms, the most frequent being flushing, diarrhea, dyspnea and wheezing, caused by bioactive amines produced by serotonin-producing neuroendocrine tumors. The most typical example is excess serotonin production by midgut neuroendocrine tumors with liver metastases. Carcinoid crisis is an extreme form of carcinoid syndrome where acute release of high amounts of hormones cause hemodynamic instability, usually in the context of tumor manipulation or anesthesia. Carcinoid heart disease is a chronic complication of serotonin excess consisting of fibrous endocardial thickening affecting the tricuspid and pulmonary valves, thereby causing either regurgitation or stenosis of these valves. It occurs in up to 50% of patients with carcinoid syndrome and correlates with elevated levels of serotonin and its metabolite 5-HIAA in 24 h urine collections [82,83,84,85].

Current guidelines recommend somatostatin analogue therapy as first line in patients with symptomatic carcinoid syndrome [12,13,14,15,16,17]. Multiple studies have shown that both octreotide and lanreotide, at standard and higher doses and at regular and shorter intervals, are effective at reducing symptoms of carcinoid syndrome such as flushing and diarrhea by more than 50% as well as decreasing levels of 24h urinary 5-HIAA [82,83,84,85]. Gradual dose increase or additional prescription of short-acting octreotide either for 2 weeks after the initial injection of the long-acting depot formulation or as needed during therapy may be required if patients are still symptomatic.

As mentioned earlier, liver resection may be considered as a management strategy for liver-predominant metastases in the absence of diffuse bilobar disease, compromised liver function, or widespread metastatic disease. In the case of symptomatic carcinoid syndrome, multiple retrospective series have shown that there may be an improvement in flushing and diarrhea [33,85,86,87,88] with surgical resection or hepatic embolization of hepatic metastases of small bowel NETs.

As a precaution to prevent carcinoid crisis, preoperative and intraoperative short-acting SSA therapy is recommended before hepatic resection of NET metastases. Typical recommended doses are 300–500 mcg intravenously or subcutaneously prior to surgery. If carcinoid crisis occurs, the mainstay of management remains intravenous short-acting octreotide, whether boluses of 500–1000 mcg or a continuous infusion of 50–200 mcg/h [12,13,14,15,16,17].

Apart from somatostatin analogue therapy and liver-directed therapy, all guidelines recommend telotristat etiprate as a possible next line of therapy for patients with uncontrolled carcinoid syndrome-induced diarrhea. Telotristat is an oral tryptophan hydroxylase inhibitor, thus a blocker of the rate-limiting step in the production of serotonin. In 2017, the TELESTAR trial [89], a large randomized three-arm controlled trial evaluating two doses of oral telotristat (250 mg and 500 mg, each taken three times daily) against placebo over a 12-week period including 135 patients with a history of carcinoid syndrome with poorly controlled diarrhea while on somatostatin analogue therapy showed that telotristat, at either dose, was associated with a statistically significant reduction in bowel movement frequency over time compared with placebo. There were too few patients with flushing to address that endpoint. Urinary 5-HIAA was also significantly decreased with telotristat.

Canadian consensus guidelines [12] and NCCN guidelines [14] also recommend interferon as a possible second-line therapy for patients with carcinoid syndrome and diarrhea that is poorly controlled with SSA therapy. In an open prospective trial, Frank et al. [90] demonstrated that the addition of alpha-interferon to octreotide has antiproliferative efficacy in a subgroup of patients with advanced metastatic disease unresponsive to octreotide monotherapy and that in terms of symptoms, interferon improved flushing and diarrhea by 40–50% in such patients.

NCCN guidelines also list pasireotide and PRRT as second-line therapies for carcinoid syndrome refractory to somatostatin analogue therapy. In a randomized controlled phase III trial in patients with carcinoid syndrome refractory to maximal doses of somatostatin analogues, long-acting pasireotide reduced diarrhea by 25% and flushing by 42%. [91]. Participants in the 177-lutetium + octreotide (treatment) arm of the NETTER-1 trial [49] showed a 39% improvement in diarrhea symptoms (vs. 23% for controls, no significant difference) and a 42% improvement in flushing (vs. 38% for controls, no significant difference). Although everolimus has not been specifically approved for symptomatic control of functional NET, it is often combined with somatostatin analogues in clinical practice based on the biochemical improvement in chromogranin A and 24 h urine 5-HIAA seen in the treatment arm of the RADIANT-2 trial [46], although it must be pointed out that improvement in carcinoid-related symptoms was not a secondary outcome measured in this trial.

Although resection of liver metastases in small bowel neuroendocrine tumors with excess serotonin production and somatostatin analogue therapy are unanimously recommended therapies for carcinoid syndrome [12,13,14,15,16,17], future avenues for research may include larger prospective studies to confirm the improvement in flushing and other carcinoid syndrome features with telotristat etiprate. This, along with the impact of everolimus, pasireotide and 177-lutetium alone and in different combinations and sequences, would better instruct how to optimally manage the morbidities and mortality of the carcinoid syndrome.

## 8. Management of Small Bowel Neuroendocrine Carcinoma

Small bowel neuroendocrine carcinoma (NEC) is a rare pathologic diagnosis where the tumor cells are poorly differentiated; NECs are typically characterized by an aggressive biology where they metastasize early and in a widespread fashion with an overall poor prognosis. In older definitions, any tumor with a Ki-67 greater than 20% (Grade 3) and an elevated mitotic count (>20 per 10 high power field) was classified as neuroendocrine carcinoma. Of note, following the updated 2017 WHO Pathology Classification, a subset of Grade 3 tumors that are still well differentiated was distinguished from their more aggressive counterparts, NECs, where the tumors are poorly differentiated and the Ki-67 index is usually higher than 55% [2]. This update was specific to pancreatic neuroendocrine tumors and this distinction was not specifically adopted in the WHO classification of small bowel neuroendocrine tumors, but a recent WHO consensus paper has reviewed the literature and pointed out that the distinction between NET and NEC should be based on tumor cell differentiation and genetic alterations that are distinct between NET and NEC [92].

Thus far, there have not been prospective trials specifically designed to guide management of small bowel NECs. Treatment guidelines are based on retrospective studies and small cell lung cancer literature (SCLC) given a similar biology [93] as illustrated by the SEER database analysis by Dasari et al. As surgery alone is rarely curative, for patients with early stage NEC and potentially resectable disease, surgical resection with adjuvant chemotherapy consisting of 4–6 cycles of etoposide + a platinum based drug (carboplatin or cisplatin) is recommended. This is consistent with NANETS guidelines for poorly differentiated NEC [94]. For locally advanced non-metastatic NEC, neoadjuvant chemotherapy with radiation and surgery, if possible, is recommended. For patients with metastatic NEC, palliative chemotherapy with etoposide + platinum-based drug (cisplatin or carboplatin) or iriotecan + cisplatin are standard regimens. As opposed to low-intermediate grade tumors, surgical metastectomy is generally not recommended in the management of NEC, although could be considered in select symptomatic patients. Second-line chemotherapy regimens include temozolomide, fluoropyrimidine, irinotecan and oxaliplatin-based regimens [95,96,97,98,99].

With regards to those Grade 3 neuroendocrine tumors that remain well differentiated, little data exists to guide their specific management. Recommendations are only expert consensus ones and overlap management strategies for Grade 1–2 tumors and NEC. For localized disease, surgical resection is reasonable. Advanced well-differentiated G3 NET respond inadequately to platinum/etoposide regimens and, therefore, it is not recommended as first line unless the disease is aggressive at diagnosis. For instance, in the NORDIC NEC study [100], a retrospective study investigating a cohort of 305 patients with Grade 3 gastrointestinal NEC showed that those with a Ki-67 less than 55% were less responsive to platinum-based chemotherapy regimens. However, they had a longer survival than those with a Ki-67 index greater than 55%. Of note, the study population of the NORDIC NEC study included both pancreatic and other gastrointestinal neuroendocrine tumors, which suggests that the biology and treatment response to chemotherapy is seen not only in pancreatic but also other (including small bowel) Grade 3 NET.

Interestingly, an ongoing randomized clinical trial (NCT02595424) comparing progression-free survival response to different chemotherapy regimens (cisplatin, carboplatin and etoposide versus capecitabine-temozolomide) in metastatic or unresectable Grade 3 small bowel or pancreatic neuroendocrine tumors is under way and should shed light on the optimal treatment for this subgroup of patients [101]. Future avenues for research should also include evaluating outcomes in well-differentiated grade 3 small bowel neuroendocrine tumors treated with systemic therapies such as somatostatin analogues, everolimus, PRRT as well as chemotherapy regimens (e.g., capecitabine-temozolomide) or a combination of these to guide clinicians in managing such challenging cases.

## 9. Conclusions

In summary, in accordance with current major guidelines and evidence, the cornerstone of management of localized small bowel neuroendocrine tumors is surgical resection of the primary tumor(s) and lymphatic drainage field. In the case of synchronous diagnosis of the primary tumor and resectable hepatic metastases, resection of the primary tumor, and liver metastases is recommended. Hepatic metastases of small bowel neuroendocrine tumors should be resected surgically or by image-guided ablation or, if impossible, treated with bland embolization, chemoembolization, or radioembolization.

For unresectable or metastatic disease, therapy with a somatostatin analogue (octreotide LAR or lanreotide) is recommended as first line option in well-differentiated, somatostatin-receptor positive disease. Second-line options include mTOR inhibitor everolimus and PRRT with 177-lutetium for SSA-refractory disease.

Carcinoid syndrome is managed mainly with long-acting somatostatin analogues chronically and short-acting somatostatin analogues can be used in prevention or management of carcinoid crisis during surgical procedures. Telotristat etiprate, a tryptophan hydroxylase inhibitor, is an interesting second-line treatment for patients with diarrhea that is refractory to somatostatin analogue therapy.

Evidence is scarce to guide management of patients with neuroendocrine carcinomas. Current recommendations are based on retrospective studies and small cell lung cancer (SCLC) literature given a similar biology. For early stage NEC and potentially resectable disease, surgical resection with adjuvant chemotherapy consisting of etoposide + a platinum-based drug is recommended.

Overall, important landmark trials have redefined management of small bowel neuroendocrine tumors in recent years, with the confirmation of the antitumor effect of somatostatin analogue therapy in PROMID and CLARINET trials as well as the advent of therapies with significant potential such as everolimus (RADIANT trials) and PRRT with 177-lutetium (NETTER-1 trial). However, several questions remain unanswered, such as the optimal effect of combining the aforementioned therapeutic modalities and possibly their sequence, and more randomized controlled trials are needed to address such management dilemmas.

## Figures and Tables

**Table 1:** Comparison of 4 guidelines on the management of small bowel neuroendocrine tumors.

Guidelines Situations	Canadian Consensus Guidelines 2016 (12)	NANETS (North American Neuroendocrine Tumor Society) Guidelines 2017 (13–14)	NCCN (National Comprehensive Cancer Network) Guidelines 2018 (15)	ENETS (European Neuroendocrine Tumor Society) Guidelines 2016 (16–17)
**Locoregional disease**	→ Complete resection of the primary tumor and the associated lymphatic drainage field → Evaluate for multifocality (*Unclear if favor open vs. laparoscopic minimally invasive*)	→ Complete open resection of primary tumor and the associated lymphatic drainage field → *Laparoscopic resection is acceptable if bowel can be completely run through a small incision*	→ Complete resection of the primary tumor and the associated lymphatic drainage field → Evaluate for multifocality (*Unclear if favor open vs. laparoscopic/ minimally invasive*)	→ Complete open (or in selected patient laparoscopic) resection of primary tumor and the associated lymphatic drainage field
**Residual disease or positive margins post-primary resection**	→ *Definitive resection when technically feasible*	→ Not addressed	→ Not addressed	→ Not addressed
**Synchronous primary and metastatic disease**	→ Resection of primary tumor(s), lymph nodes in combination with liver metastases	→ Resection of primary tumor(s), lymph nodes in combination with liver metastases	→ Resection of primary tumor(s), lymph nodes in combination with liver metastases	→ Local radical open resection of primary tumor(s), lymph nodes in combination with liver metastases
**Liver metastases**	→ Resection of liver metastases with the goal of *preserving liver parenchyma and both left and right inflow and outflow vascular patency when possible*. → Image-guided ablation either alone for limited disease (tumors ideally < 3 cm) or in combination with surgery → If cytoreductive surgical/ablative procedures not indicated; bland embolization, chemoembolization or radioembolization	→ Resection of liver metastases should be attempted when feasible and low morbidity/mortality → Parenchymal sparing procedures should be considered → Patients with any number or size of metastases, intermediate grade, extrahepatic disease should be considered for liver debulking operations *if a 70% debulking threshold can be achieved*. → If cytoreductive surgery not indicated; bland embolization, chemoembolization or radioembolization	→ Resection of liver metastases or ablative therapies such as RFA (Radio-Frequency Ablation) or cryoablation may be considered if near-complete treatment of tumor burden can be achieved. → If cytoreductive surgical/ablative procedures not indicated; bland embolization, chemoembolization or radioembolization	→ Resection of liver metastases when feasible → Image-guided ablation can be combined with surgical resection → If cytoreductive surgical procedures not indicated: radio-frequency ablation, laser-induced thermotherapy, transarterial chemoembolization, transarterial embolization or selective internal radiation therapy (investigational)
**Peritoneal metastases**	→ Surgical resection when feasible and synchronous resection of peritoneal disease and hepatic metastasectomy is an option.	→ Surgical resection when feasible → No evidence supporting use of HIPEC (hyperthermic intraperitoneal chemotherapy)	→ Not addressed	→ Not addressed
**Abdominal disease in setting of extra-abdominal metastases**	→ *Cytoreduction should be considered in selected patients for symptom control*	→ Not addressed	→ Not addressed	→ Not addressed
**Prophylactic cholecystectomy**	→ *Consider as part of any abdominal surgical procedure*	→ If future treatment with SSA is anticipated, a prophylactic cholecystectomy can be considered	→ If future treatment with SSA is anticipated, a prophylactic cholecystectomy can be considered	→ If future treatment with SSA is anticipated, a prophylactic cholecystectomy can be considered
**Systemic therapy for metastatic or unresectable disease**	→ First line: somatostatin analogues (octreotide LAR (Long Acting Release), lanreotide autogel) → Second line: everolimus single agent OR everolimus + SSA OR PRRT (Peptide Radionuclide Radiation Therapy)	→ First line: somatostatin analogues (octreotide LAR, lanreotide autogel) → Second line: PRRT (preferred) OR everolimus → *Other less favored modalities: Interferon alpha 2b or locoregional therapy*	→ First line: somatostatin analogues (octreotide LAR, lanreotide autogel) or watch and wait if asymptomatic with low tumor burden → Second line: start SSA if prior watch and wait OR everolimus OR PRRT OR *locoregional therapy OR interferon alpha 2b OR cytotoxic chemotherapy if no other options feasible*	→ First line: somatostatin analogues (octreotide LAR, lanreotide autogel) or watch and wait if G1, low tumor burden, stable and asymptomatic → Second line: Start SSA if prior watch and wait OR locoregional therapy OR PRRT OR everolimus *OR interferon alpha 2b*
**Symptom control—Carcinoid syndrome**	→ First line: somatostatin analogues (octreotide LAR, lanreotide Autogel) → Short-acting octreotide recommended for two weeks after first long-acting SSA injection → Refractory to SSA: telotristat etiprate OR interferon alpha OR SSA dose escalation	→ First line: somatostatin analogues (octreotide LAR, lanreotide autogel) → Short-acting octreotide recommended for two weeks after first long-acting SSA injection → Refractory to SSA: telotristate etiprate	→ First line: Somatostatin analogues (Octreotide LAR, Lanreotide Autogel) → Short-acting octreotide recommended for two weeks after first long-acting SSA injection → Refractory to SSA: Telotristate etiprate	→ First line: somatostatin analogues (octreotide LAR, lanreotide autogel) → Refractory to SSA: SSA dose increase OR add-on interferon alpha 2b OR *pasireotide OR PRRT* OR telotristat etiprate
**Symptom control—Bone and brain metastases**	→ *External beam radiotherapy is an option*	→ Not addressed	→ Not addressed	→ Not addressed

→ Major differences between guidelines are italicized in Table 1.

**Table 2:** Landmark trials in the treatment of advanced or metastatic neuroendocrine tumors (NETs).

Trial	Eligibility Criteria	Intervention	n	Median PFS	Adverse Effects	Health-Related Quality of Life
**PROMID (8)** **2009**	Treatment-naïve midgut NETs, secretory/NS	Octreotide LAR 30 mg IM q4w vs. Placebo IM q4w	85 (42 vs. 43)	Median Time-to-Tumor-Progression 14.3 mo vs. 6.0 mo HR = 0.34 (0.2–0.59), *p* 0.000072	Any AES: 26 vs. 23 Hematological AES: 12 vs. 2 Fatigue and fever: 19 vs. 5	**PROMID HRQoL Study (48)** Time to definitive deterioration (OC vs. PBO) - 18.5 mo vs. 6.8 mo (fatigue) - NR vs. 18.2 mo (pain) - NR vs. 16.4 mo (insomnia) NR: Not reached
**CLARINET (9)** **2014**	Previous treatment permitted, enteropancreatic NETs, non-secretory, SSR+	Lanreotide Autogel 120 mg dsq q4w vs. Placebo sc q4w	204 (101 vs. 103)	NR vs. 18 mo HR = 0.47 (0.30–0.73), *p* < 0.001	Diarrhea: 26 vs. 9 Abdominal pain: 14 vs. 2 Any AES: 25 vs. 31	No HRQoL study
**RADIANT-4 (10)** **2016**	Advanced lung or GI NETs, nonsecreting, prior treatment allowed	Everolimus 10 mg po die vs. Placebo	302 (205 vs. 97)	11 vs. 3.9 mo HR 0.48 (0.35–0.67), *p* < 0.00001	Stomatitis: 63 vs. 19 Diarrhea: 31 vs. 16 Infections: 29 vs. 4 Rash: 27 vs. 8 Peripheral edema: 26 vs. 4 Anemia: 16 vs. 2 Anorexia: 16 vs. 6 Asthenia: 16 vs. 5 Pneumonitis: 16 vs. 1 Dysgueusia: 15 vs. 4 Cough: 13 vs. 3 Stomatitis: 8 vs. 0 Diarrhea: 7 vs. 2	**RADIANT-4 HRQoL Study (49)** Time to definitive deterioration (FACT-G global score) (EV vs. PBO) 11.27 mo vs. 9.23 mo (AHR 0.81, *p* 0.31)
**NETTER-1 (11)** **2015**	NETs progressing on Octreotide LAR (30 mg), midgut NETs, secretory1nonsecretory, SSR +	7.4 GBq 177-Lu-Dotatate q8w + Octreotide LAR 30 mg IM q4w vs. Octreotide LAR 60 mg IM q4w	229 (116 vs. 113)	NR vs. 8.4 mo HR = 0.209 (0.129–0.388), *p* < 0.0001	Any AES: 86 vs. 31	**NETTER-1 HRQoL Study (50)** Time to definitive deterioration (PRRT vs. PBO) Longer - Global health status 28.8 mo vs. 6.1 mo (HR 0.406) - Physical functioning 25.2 vs. 11.5 mo (HR 0.518) - Role functioning (HR 0.580) - Fatigue (HR 0.621) - Pain (HR 0.566) - Diarrhea (HR 0.473) - Disease-related worries (HR 0.572) Body image (HR 0.425)
